# Design and Development of Mixed Film of Pectin: Ethyl Cellulose for Colon Specific Drug Delivery of Sennosides and *Triphala*

**DOI:** 10.4103/0250-474X.42998

**Published:** 2008

**Authors:** Munira Momin, K. Pundarikakshudu, S. A. Nagori

**Affiliations:** L. J. Institute of Pharmacy, Sanand Cross Road, Sarkhej Gandhinagar Highway, Ahmedabad-382 210, India; 1 L. M. College of Pharmacy, Navarngpura, Ahmedabad-380 009, India

**Keywords:** Sennosides, *Triphala*, pectin, ethyl cellulose, film coating, colonic drug delivery

## Abstract

The present study was aimed at developing colon specific drug delivery system for sennosides and * Triphala*. These drugs are reputed Ayurvedic medicines for constipation in India. The proposed device explored the application of pectin and ethyl cellulose as a mixed film for colon specific delivery. This mixed film was prepared using non-aqueous solvents like acetone and isopropyl alcohol. A 32 factorial design was adopted to optimize the formulation variables like, ratio of ethyl cellulose to pectin (X1) and coat weight (X2). The rate and extent of drug release were found to be related to the thickness and the ratio of pectin to ethyl cellulose within the film. Statistical treatments to the drug release data revealed that the X1 variable was more important than X2. Under simulated colonic conditions, drug release was more pronounced from coating formulations containing higher proportions of pectin. The surface of the device was coated with Eudragit S100 to ensure that the device was more pH dependent and trigger the drug release only at higher pH. The final product is expected to have the advantage of being biodegradable and pH dependant. This type of a film effectively releases the drug while maintaining its integrity.

Constipation is a very complicated problem. Although only a few options are available via allopathic drugs, safe and effective drugs are available from herbal origin. Senna is a well-known drug in Ayurvedic system of medicine containing anthraquinone glycosides known as sennosides A and B. The action of sennosides is mainly on the large intestine and therefore, especially suitable in habitual constipation^1^–^3^. *Triphala* is a traditional Ayurvedic herbal formulation for constipation, consisting pericarp from fruits of three medicinal plants, *Amla* (*Embelica officinalis,* Family: Euphorbiaceae), *Harade* (*Terminalia chebula,* Family: Combretaceae) and *Baheda* (*Terminalia belierica,* Family: Combretaceae) in equal proportions^4^–^5^. One can expect optimal result, if the drugs are targeted directly to the colon. We reported colon targeted delivery systems containing either calcium sennosides or *triphala* extract^6^–^9^. A comprehensive formulation containing *triphala* extract with sennosides is expected to make the formulation more logical and beneficial as compared to the ingredients as single drug. Hence an attempt was made to incorporate both sennosides and *triphala* extracts, in a colon targeted delivery system.

The basic approaches to achieve colonic site specificity include utilizing concept of pH changes in the gastro intestinal region and designing a system that releases the drug at a predetermined time after administration. Several polysaccharides like, pectin and its salts, chondroitin sulphate, amylose and guar gum are being investigated as carriers for colon specific drug delivery 10–15. Pectin is one such heterogenous polysaccharide consisting mainly of D-galacturonic acid and its methyl ester linked via α(1-4) glycosidic bonds. Various reports suggest that pectin and its salt may be of value in designing drug delivery formulation to the colon^16^–^17^. The combination of this type of biodegradable polymer with a film former has been investigated recently^18^–^19^. Due to their high solubility and swelling properties in aqueous media, film coatings prepared with pectin or its salts alone are unable to prevent the release of drugs from coated dosage forms during their transit through the stomach and the small intestine. These mixed coatings can prevent the swelling and the solubilization of pectin during the transit from mouth to caecum. On the other hand, the degradation of pectin by the pectinolytic enzymes of the colonic flora is expected to increase the drug release in the colon by the formation of a more porous coating structure.

Most of the research has been done with aqueous base pectin film coating systems. In the present study an effort was made to develop a pectin-ethyl cellulose coating system using non aqueous solvent. Here, we report film coated colon targeted drug delivery system containing calcium sennosides and *triphala* in combination using pectin and ethyl cellulose as film former. This non-aqueous condition would involve lower temperature and shorter processing time, conditions that may be favorable to water and/or temperature sensitive drugs. A 3^2^ full factorial design was employed in this study to systematically design and develop colon specific formulation.

## MATERIALS AND METHODS

Powders of *Amla, Harade* and *Baheda* were purchased from an established local supplier, L. V. Gandhi and Sons, Ahmedabad and passed through 60 meshes. Rectified spirit was used for preparation of *triphala* extracts. Calcium sennoside (20%w/w) was gifted from Dishman Pharmaceuticals, Ahmedabad, India. Pectin USP, ethyl cellulose (EC), Eudragit S 100, cross carmillose sodium, co-processed lactose-microcrystalline cellulose (co-processed lactose-MCC) and polyvinyl pyrrolidone (PVP K30) were gifted from Zydus Cadila Healthcare Ltd, Ahmedabad, India. Ethyl alcohol, acetone, dichloromethane, dibutyl phthalate, castor oil were of a reagent grade and used as obtained. Pectinex was obtained from Biocon India Limited, Banglore, India. All other chemicals were of reagent grade.

### Preparation of calcium sennoside and *Triphala* uncoated tablets:

Calcium sennoside (100 mg, 20%w/w sennosides calculated as sennoside B) and *triphala* extracts (300 mg) were mixed with co-processed lactose-MCC, cross carmillose sodium (3%w/w) and PVP K30 (2% w/w). Powder blend was sieved through 60# sieve and directly compressed using a rotary tablet press (Cadmach Machinery, India) having 12 mm size concave die. Weight variation, crushing strength; friability, thickness, disintegration time and dissolution in phosphate buffer pH 7.4 were performed for the uncoated tablets. The average tablet weight was 600±7 mg. Tablet had a crushing strength and friability of 5.4 kgf and 0.21%, respectively.

### Preparation of coating formulations:

Pectin and EC were used in different proportion as per 3^2^ factorial designs (Table 1). A measured quantity of pectin was first added to a mixture of acetone and isopropyl alcohol (40:60) and mixed using magnetic stirrer for 20 min. The plasticizer (dibutyl phthalate) was added to the solution based on the solid dry weight (12% w/w) of pectin present and mixed for 30 min. The required quantity of EC was dissolved in ethyl alcohol containing 7%w/w (based on the solid dry weight of EC) propylene glycol as plasticizer. This plasticized ethyl cellulose solution was then added to non-aqueous pectin solution and stirred for another 30 min using magnetic stirrer to produce coating formulation.

**TABLE 1:** 3^2^ FULL FACTORIAL DESIGN FOR SENNOSIDE AND *TRIPHALA* COLON TARGETED TABLETS

Batches	X1	X2	Y360	
			
			Sennosides	*Triphala*
TS1	-1	-1	100.04	98.6
TS2	-1	0	100.1	98.2
TS3	-1	1	98.7	97.2
TS4	0	-1	96.1	95.2
TS5	0	0	86.5	85.3
TS6	0	1	78.3	77.8
TS7	1	-1	71.5	70.0
TS8	1	0	63.1	61.8
TS9	1	1	42.5	41.6

Y360 is drug released after 360 minutes, X1 is percentage of pectin replaced by ethyl cellulose and X2 is coat weight over tablet as % w/w. Translation of coded levels in actual units was for X1, -1, 0 and 1 correspond to 20, 35 and 50, and for X2, -1, 0 and 1 correspond to 5, 7 and 10, respectively

### Experimental design:

A 3^2^ full factorial design was utilized in the present investigation^20^. The pectin:ethyl cellulose ratio in coat (X1) and coat weight (X2) were used as independent variables. The experimental design is presented in Table 1. The chosen dependent variables were Y360 (percent drug release after 360 min in presence of pectinolytic enzyme)

### Film coating process:

The coating of sennosides and *triphala* tablet was done by conventional rotating pan at 35 rpm. The coating procedure involved maintaining the bed temperature at 25° to 28°. The desired volume of the coating solution was sprayed on pre-warmed tablet (batch size 60 g) bed in a pan coater. The tablets were coated and dried with the help of inlet air having temperature 35° to 40°. The coating procedure was repeated till the desired level of coating was achieved (Table 1). The percentage mass increase of the tablet upon coating was taken to be indicative of the coat thickness. The final drying stage was done by stopping the spraying of coating solution and keeping the coated tablets at the same bed temperature for 20 min.

The optimized formulation was coated with Eudragit S100 to ensure the device more pH dependent and trigger the drug release only at higher pH. Eudragit S100 (10% w/v) was dissolved in isopropyl alcohol containing 3% plasticizer (polyethylene glycol-400). The coating solution was sprayed with the help of inlet air having temperature 35° to 40°. The coating procedure was repeated till the coat weight increased to 5% w/w of original weight of table. Rest of the coating conditions were kept same as that of with pectin EC coating.

### *In vitro* drug release studies:

The ability of coat applied on core tablets to remain intact in the physiological environment of stomach and small intestine was assessed by mimicking mouth to colon transit. Drug release studies were carried out using USP dissolution apparatus (Apparatus II, 100 rpm, 37± 0.5°) for 2 h in 900 ml, 0.1 N HCl^21^. Then, the dissolution medium was replaced with 900 ml PBS (phosphate saline buffer pH 7.4), and the dissolution was continued for another 3 h. Ten millilitres of the sample was taken at the end of the specified time period (2, 5, 6, 8 and 10 h) and analyzed for sennosides and total tannin content as described below. A 10 ml volume of fresh and filtered dissolution medium was added to make the volume after each sample withdrawal. The susceptibility of the coat to the enzymatic action of colonic bacteria was assessed by continuing the drug release studies in simulated colonic fluids prepared by adding 4% w/v Pectinex^®^.

### Determination of sennosides and total tannin content of tablets:

After each interval of dissolution study, 10 ml sample was withdrawn and analyzed for sennosides and total tannins content. Sample was first shaken with ethyl acetate. Aqueous part containing sennosides are analyzed as per BP method^22^. Ethyl acetate part containing tannins is dried; residues are dissolved in distilled water and analyzed for total tannins content as per AOAC method^23^.

## RESULTS AND DISCUSSION

A number of factors are important for oral colon specific drug delivery system. One of the factors for selection of an approach is solubility of the drug. Matrix tablets of water-soluble drug may release a significant amount of drug from the matrix surface in the physiological environment of stomach and intestine before reaching at the site of action. In such a situation either the tablets can be enteric coated or application of compression coat will retard the drug release in gastrointestinal tract. Calcium sennosides and *triphala* are water-soluble drugs. Hence, an attempt was made to minimize the drug release in the physiological environment of gastrointestinal tract and to ensure maximum drug release in colon by applying mixed film of pectin and ethyl cellulose.

Nine batches of tablets containing sennosides and *triphala* were satisfactorily coated using non-aqueous solvents based coating system. The non-aqueous system is particularly suited to coating the drugs that are sensitive to water and/or heat. Preliminary study revealed that combination of dibutyl phthalate and propylene glycol when used as plasticizer gives better film characteristics. A 3^2^ full factorial design was applied to optimize the film coated sennosides- *triphala* formulation using pectin and EC. The responses were measured and polynomial equations were derived by carrying out multiple regression analysis and F-statistics to identify statistical significance. Y= b_0_ + b_1_X_1_ + b_2_X_2_ + b_12_X_1_X_2_ + b_11_X_1_^2^ + b_22_X_2_^2^ (Eqn.1)

In Eqn. 1, Y is a dependent variable, and b_0_, b_1_, b_2_, b_12_, b_11_, b_22_ are constants or coefficients of the various terms in the equation. The main effects (X_1_ and X_2_) represent the average results of changing one factor at a time from its low to high value. The interaction (X_1_ X_2_) shows how the response changes when two factors are changed simultaneously. The polynomial terms (X_1_^2^ and X_2_^2^) are included to investigate non-linearity. Thus, a systematic study was adopted by applying 3^2^ full factorial designs. The proportion of pectin and EC in coat (X_1_) and coat weight (X_2_) were used as independent variables. To elucidate the influence of coating thickness and pectin EC ratio on drug release, following polynomial equation was evolved. Here, percent drug released after 6 h of dissolution of tablet in presence of pectinolytic enzyme, Pectinex is used as response. Y360 (sennosides) = 86.65-19.87X_1_-7.6X_2_-3.14X_1_X_2_-5.13X_1_^2^+0.471X_2_^2^ (Eqn. 2), (R^2^= 0.995, F= 82.96, DF= 7). Y360 (*Triphala*) = 86.44-18.85X_1_-6.616X_2_-4.875X_1_X_2_-6.951X_1_^2^+0.451X_2_^2^(Eqn. 3), (R^2^- 0.993, F- 91.236, DF- 5).

**Fig. 1:**
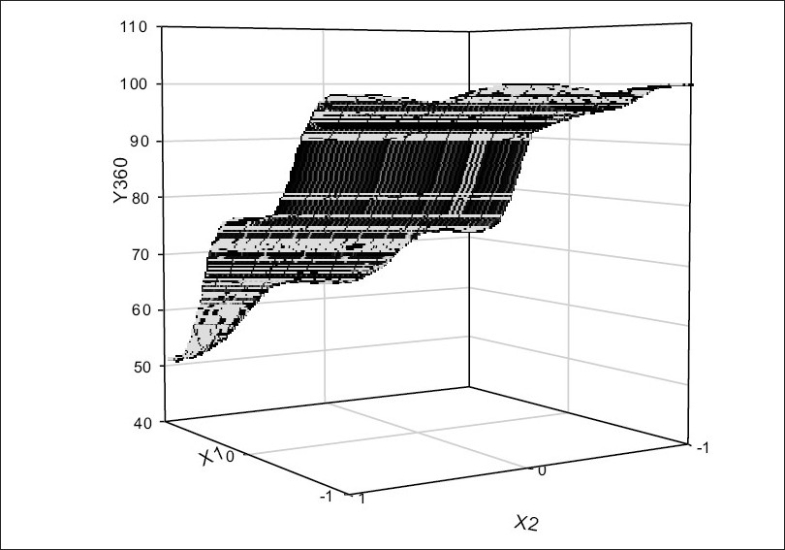
Response surface plot for sennosides Y360 is drug released after 360 minutes, X1 is percentage of pectin replaced by ethyl cellulose and X2 is coat weight over tablet as % w/w.

The value of Y360 (sennosides) for the nine batches showed a wide variation from a minimum of 51.3% to a maximum of 100.04%, indicating that the independent variables influence the selected dependent variable. The values for Y360 (*Triphala*) ranged from 49.1 and 98.6%. Eqns. 2 and 3 shows that the pectin EC ratio (X1) has more influence on drug release than the coating thickness (X2). All the terms in Eqns. 2 and 3 were found to be significant and summarized in Table 2. Grid search technique and/or response surface plot may be used to find optimum combination of X_1_ and X_2_ or the trend. (I.e. how the response changes with change in X_1_ and X_2_) in the measured response (fig. 1).

**TABLE 2:** REGRESSION OUTPUT FOR SENNOSIDES AND *TRIPHALA*

			Regression output	
			
			Sennosides	*Triphala*
R squared			0.996	0.993
Multiple R			0.998	0.996
No. of observation			8	9
Degree of freedom			5	5

	X Coefficient		P value	
		
	Sennosides	*Triphala*	Sennosides	*Triphala*

b0	86.65	86.44	0.000	0.00009
b1	-19.87	-18.85	0.004	0.0003
b2	-7.605	-6.616	0.028	0.0064
b12	-3.143	-4.875	0.214	0.2634
b12	-5.129	-6.951	0.11	0.0257
b22	0.471	0.451	0.823	0.8065

b_0_, b_1_, b_2_, b_12_, b_11_, b_22_ are coefficients of the various terms in the polynomial equation.

**Fig. 2:**
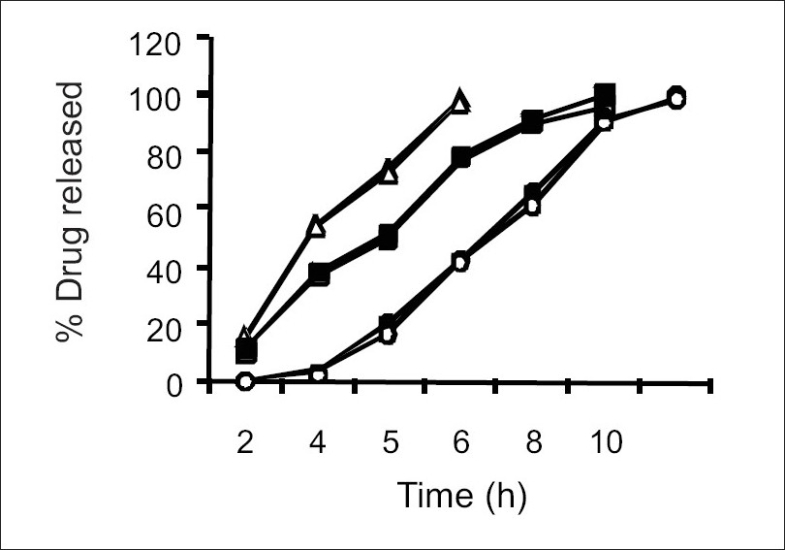
Effect of ethyl cellulose levels on Triphala and Sennosides releases EC is ethyl cellulose, S is sennosides and T is Triphala; Formulation containing 20 % EC (sennosides —▲—, Triphala —Δ—), formulation containing 35% EC (sennosides —■—, Triphala —□—), formulation containing 50% EC (sennosides —◆—, Triphala —◇—)

**Fig. 3:**
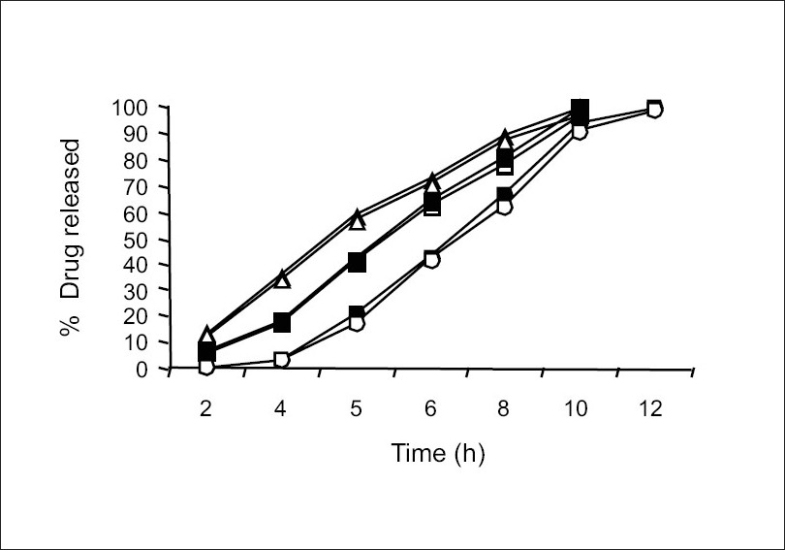
Effect of coating levels on Triphala and Sennosides releases S is sennosides and T is triphala. Formulation with 5% coat weight (sennosides —▲—, triphala —Δ—), formulation with 7% coat weight(sennosides —■—, triphala —□—), formulation with 10% coat weight (sennosides —◆—, triphala —◇—)

For the drug delivery system designed for colon targeting, it is desirable that the system remains intact in the physiological environment of stomach and upper intestine and releases the drug in the colon. For the present investigation it is desirable to design the formulation such that it releases sennosides and *triphala* content in colon only without loss of drug in the upper GI tract. The film coat was designed to undergo bacterial degradation in the colon. To determine the bacterial degradation of pectin EC coat, dissolution studies were carried out with and without 4% w/v Pectinex, which was added at 4 h to simulate the colon arrival time under normal conditions. As per the experimental design of sampling intervals sennosides and total tannin was detected for all the batches. Y360 of all batches are shown in Table 1. Dissolution was continued up to 10 h depending on the tablet degradation pattern. Comparative dissolution profiles of all the batches are shown in Figs. 2 and 3. The dissolution profiles indicate that the drug release is directly proportional to the amount of pectin present in film and inversely proportional to thickness of the film. At lower level of X1, premature drug release was observed. Batches 1 to 3 showed Y360 of 100.04, 98.7 and 96.1 of sennosides with enzyme in dissolution medium. Almost similar results were observed in *triphala* release. In batches 1 to 3 proportion of EC is less. Hence the film containing higher proportion of pectin swell in presence of aqueous medium and forms water permeable pores through which diffusion of drug occurs. Film coating containing higher proportion of pectin is therefore more permeable for release of sennosides and *triphala*. One of the key requirements of colon specific drug delivery system is, it must delay the drug release until it passes through the upper gastrointestinal tract. The 6 h dissolution test used in these studies should be sufficient to assess this, since the mouth to colon transit time of tablet dosage forms have been found to be of this order. Tablets with thick coat showed slower drug release. Equation 2 and 3 shows that the pectin EC ratio (X1) has more influence on drug release than the coating thickness (X2). Hence, a formulation with a thicker film coat and lower pectin content appears to comply with the requirement of colon targeting of drug. Higher EC levels (batch TS9) show slower drug release. Y360 value was found to be 42.5% and 41.6% for sennosides and *triphala,* respectively. This indicates the drug release is very slow due to insoluble ethyl cellulose, which could not be degraded by bacterial enzymes. Hence, Batch TS9 was selected as optimized formulation as the Y360 is minimum. Batch TS9 appears to be most promising batch.

**Fig. 4:**
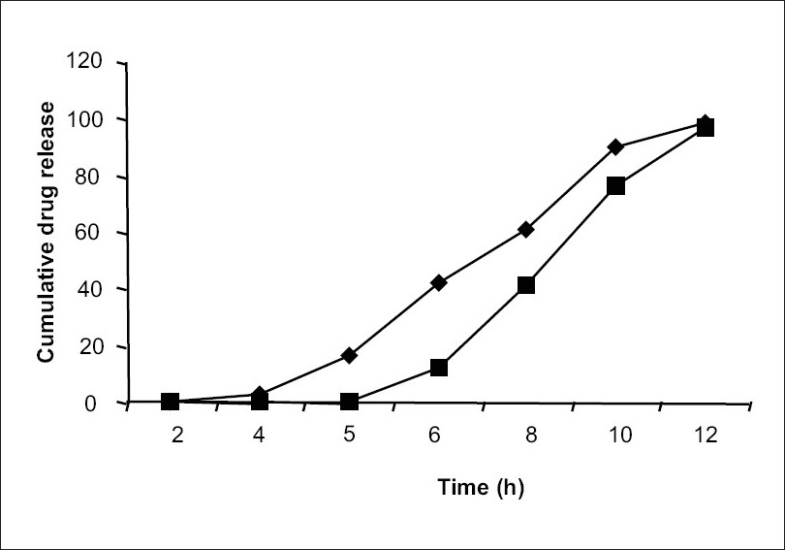
Effect of enteric coating on release of sennosides EC is ethyl cellulose; sennosides release from EC pectin coated tablet (—◆—), sennosides released from formulation with second coat of Eudragit S100 (—■—)

All the formulations were subjected to dissolution study with and without pectinex in the dissolution medium. A significant difference (P< 0.001) was observed in the amount of sennosides and total tannin released from above formulations when compared to dissolution study without Pectinex. Further to make the device more pH dependent and to trigger the drug release only at higher pH, uppermost coating layer with Eudragit S100 was given to the optimized batch formulation. Comparative dissolution pattern is shown in Fig. 4. Hence, it can be concluded that if Eudragit S100 is used as final coating layer it gives promising results.

From the overall dissolution data it can be concluded that EC plays a major role in optimization of the formulation, coat thickness helps in protecting core tablet up to 6 h and addition of pectinex, a proteolytic enzyme in dissolution media is essential to mimic the colon environment. The final product has the advantage of being biodegradable and pH dependant. This type of a coating effectively releases the drug while maintaining film integrity.
